# Better quality score compression through sequence-based quality smoothing

**DOI:** 10.1186/s12859-019-2883-5

**Published:** 2019-11-22

**Authors:** Yoshihiro Shibuya, Matteo Comin

**Affiliations:** 1 Department of Information Engineering, University of Padova, via Gradenigo 6/A, Padova, Italy; 2 Laboratoire d’Informatique Gaspard-Monge (LIGM), University Paris-Est Marne-la-Vallée, Bâtiment Copernic - 5, bd Descartes, Champs sur Marne, France

**Keywords:** FASTQ compression, BWT, FM-Index

## Abstract

**Motivation:**

Current NGS techniques are becoming exponentially cheaper. As a result, there is an exponential growth of genomic data unfortunately not followed by an exponential growth of storage, leading to the necessity of compression. Most of the entropy of NGS data lies in the quality values associated to each read. Those values are often more diversified than necessary. Because of that, many tools such as Quartz or GeneCodeq, try to change (smooth) quality scores in order to improve compressibility without altering the important information they carry for downstream analysis like SNP calling.

**Results:**

We use the FM-Index, a type of compressed suffix array, to reduce the storage requirements of a dictionary of k-mers and an effective smoothing algorithm to maintain high precision for SNP calling pipelines, while reducing quality scores entropy.

We present YALFF (Yet Another Lossy Fastq Filter), a tool for quality scores compression by smoothing leading to improved compressibility of FASTQ files. The succinct k-mers dictionary allows YALFF to run on consumer computers with only 5.7 GB of available free RAM. YALFF smoothing algorithm can improve genotyping accuracy while using less resources.

**Availability:**

https://github.com/yhhshb/yalff

**Electronic supplementary material:**

The online version of this article (10.1186/s12859-019-2883-5) contains supplementary material, which is available to authorized users.

## Introduction

Modern sequencing technologies produce large amount of data compared to the older machines. A single run can produce dozens of gigabytes, but in the near future the amount of data is going to grow in the orders of terabytes [1]. This poses the serious question of how to efficiently store and transmit these huge data sets, especially in anticipation of widespread adoption of personalized medicine and machine learning tasks.

The preferred files in which data are stored by sequencers is the well known FASTQ format. It is a textual file containing, for each read, an identifier, the nucleotide sequence, and a quality string. The quality string has the same length as the nucleotide sequence and each character encodes the probability of error of the corresponding base. The probability is usually encoded using the Phred quality score system [2]. Quality values are often essential for assessing sequence quality, filtering out low-quality reads, mapping reads to a reference genome, assembling genomic sequences, detecting mutations for genotyping, reads clustering [3, 4] and comparison [5].

To reduce the memory required by a FASTQ file it is necessary to compress it. The DNA compression is usually as simple as assigning a two bit encoding to each of the four bases. This encoding achieve almost similar results to standard lossless compressors [6]. Moreover, the sequence exposes a high redundancy, especially on large reads collections with high coverage, and a number of methods have been developed to compress it [7–10]. On the other hand, the quality values span a wider range of values, and when compressed they can sum up to about 70% of the total space to encode a FASTQ file [11].

Quality scores are more difficult to compress due to a larger alphabet (63-94 in original form) and intrinsically have a higher entropy [12]. With lossless compression algorithms and entropy encoders reaching their theoretical limits and delivering only moderate compression ratios [13], there is a growing interest to develop lossy compression schemes to improve compressibility further.

To further reduce the file sizes, Illumina proposed a binning method to reduce the number of different quality values from 42 to 8 [14]. With this proposal, Illumina opened the doors for allowing lossy compression of the quality values. Another approach called P-Block [15] involves local quantization so that a representative quality score replaces a contiguous set of quality scores that are within a fixed distance of the representative score. Similarly, the R-Block [15] scheme replaces contiguous quality scores that are within a fixed relative distance of a representative score. Other lossy approaches improve compressibility and preserve higher fidelity by minimising a distortion metric such as mean-squared-error or L1-based errors (Qualcomp and QVZ) [6, 16]. The drawback of lossy compression of quality values is that downstream analyses could be affected by the loss incurred with this type of compression. This could be the case for the above methods that process only the string of quality scores, without considering the DNA sequence associated to the read. However, [12, 17] and [11] showed that quality values compressed with more advanced methods could achieve not only a better performance in downstream analyses than Illumina-binned quality values, but even better performance than the original quality values in some cases because these methods remove noise from the data.

The most promising methods are those using both sequence and quality information. The first method proposed in this class is [18], where the authors applied the Burrows-Wheeler Transform to the reads collection in order to detect groups of suffixes starting with the same prefix (with size at least k). All quality values in a group are smoothed with the mean value. Leon [19] constructs a reference from the input reads in the form of a bloom filter compressed de-Bruijn graph and then maps each nucleotide sequence as a path in the graph. If a base is covered by a sufficiently large number of k-mers from the reference its quality is set at a fixed high value. Among the most interesting tools, Quartz [12, 20], similarly to Leon, relies on an external reference to decide if a given nucleotide is wrong or not. This reference database is implemented as list of k-mers stored explicitly, that requires 24GB when gzipped. Similarly, GeneCodeq [11] also has a list of k-mers as ground truth, but the algorithm involved during smoothing is more complex than Quartz. Each base has its associated error probability recalculated using a Bayesian framework and the smoothing takes place only if the new quality is greater than the old one. Both Quartz [12] and GeneCodeq [11] require a machine with at least 32GB of RAM, because of the size of the reference database.

In this paper we present YALFF (Yet Another Lossy Fastq Filter), a reference-based quality score compressor based on k-mers and the Burrows-Wheeler Transformation (BWT) [21], that is capable to improve compression while introducing low distortion into the processed data. One of the novelties of YALFF is that, thanks to the efficient data structure (BWT), it requires only a small amout of RAM (about 5GB) and it can be run on regular laptop. In the following sections we will present YALFF, and the results of our experiments, discussing the performances of YALFF under different metrics.

## Methods

In order to compress quality values it is important, not only to process the quality scores, but also to consider the corresponding sequence of DNA associated in the read. As already demonstrated by a number of studies (see above), the sequence can be used to predict the correctness of a base, without the need of costly alignments of the reads to a reference. Instead, the use of fast alignment-free methods, mostly based on k-mers, has replaced alignment-based methods in a number of different applications of sequence comparison [22–25]. In the context of quality compression, the use of alignment-free methods have attracted the attention for the good genotyping performance [11, 12, 18, 19, 26]. These methods are based on the idea that the correctness of a base can be predicted by the context of bases that are next to it. In [18, 19] this local sequence context is computed from the input reads, using the BWT [18] or the de-Bruijn graph [19]. Instead, Quartz [12] and GeneCodeq [11] does not need to preprocess the reads, but they are based on an external dictionary of *k*-mers. In this paper we introduce YALFF that uses a similar approach to Quartz and GeneCodeq, relying on a dictionary of k-mers in order to assess if one base of a read is correct or not. The most distinctive aspect of our approach is the compression of the k-mer list using a succinct data structure which allows us to store the whole dictionary in linear space. This task is achieved by using well-known data structures and algorithms such as the BWT [21] and its implementation found in BWA [27, 28]. The main idea is to represent the list of k-mers as a single string so as to eliminate most of the redundancy in a typical k-mer dictionary. Similarly to the other methods, in YALFF the compression of quality values is performed by searching k-mers of the reads into the dictionary. The main difference is that YALFF requires all k-mers covering the base under investigation to be found in the dictionary in order to compress the corresponding quality, whereas for previous algorithms it is enough only one shared k-mer.

### BWT Indexing of k-mers Dictionary

The most common procedure to obtain a reference list of k-mers from a set of sequences is by a k-mer counting procedure. Most k-mer counters keep track of each k-mer using hash tables, which usually require huge amounts of memory even though there exist optimized implementations [29] that allows for reduced memory overhead per key stored and concurrent access. Even if the under-represented k-mers and all the counters are removed from the resulting list, it still requires a huge amount of memory. For example, the 2 bit encoded dictionary for Quartz [12] sums up to 25 GB of space. Similarly, GeneCodeq [11] extracts all k-mers from the human reference genome and store them in a dictionary. Again, the memory requirements of GeneCodeq is of 24GB of RAM. Thus, both these methods are not suited to run on small machines.

The main insights in order to reduce the size of the dictionary is that most of the information carried by a k-mer stored explicitly is redundant. This intuition is easily explained by recalling the k-mer counting procedure itself. All the k-mers counted comes from a set of reference sequences and the counting procedure is only necessary to remove the wrong ones. There is no need to keep the k-mers explicitly stored to answer simple yes/no queries over their set. Given two consecutive k-mers it is possible to reassemble them into a single (k+1)-mer thus reducing the storage requirement by k-1 bases. This reassembly step can be carried out on the k-mers dictionary of Quartz, as well on the k-mers dictionary of GeneCodeq, leading to a linear sequence, or set of sequences, that contains all the input k-mers. However, if we want to use all the k-mers of a given reference genome, there are more efficient data structures to do so.

The problem of indexing a reference genome in minute space, while providing full search capability, has been widely studied and efficient data structure are now available. The data structure chosen for this purpose is the FM-Index [30, 31] which is based on the Burrows-Wheeler transform (BWT) of a sequence. The FM-index, and its variants, are now at the basis of many algorithms in the field of sequence analysis. For example, one of the most used tool for reads mapping, BWA [32], is based on the FM-index and it requires as input the FM-index of the reference genome. For this reason, the FM-index of many genomes are available already as they are routinely used by bioinformaticians. Thus, we decided to use the FM-index of the human reference genome. Because a reference genome is also basic resource for every bioinformatician, this method has the collateral advantage of not requiring a separate indexed FASTA for compression instead sharing the same index for reads alignments.

The FM-index will be used to search for k-mers. The procedure to retrieve the position of a k-mer is the enhanced *backward search* algorithm described in [27], that is also able to account for mismatches. In our case we will search if a k-mer is present in the reference genome with up to one mismatch.

### Quality Score Smoothing

The basic idea is that a read is represented by its constituent k-mers. Then, these k-mers can be used to assess if a given base of the read is correct. If a base is predicted to be correct, then we don’t need to store the corresponding quality value, but it can be substituted with a default value indicating a base with high probability to be correct.

**Fig. 1:**
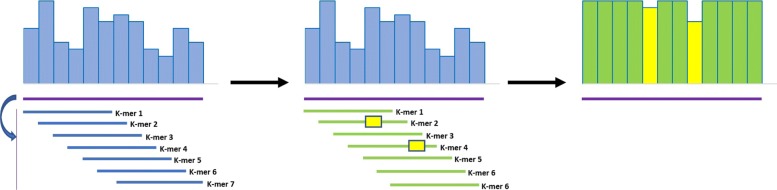
Example of smoothing performed by YALFF. A mismatch in one of the k-mers is enough to keep the corresponding quality value unchanged

The smoothing strategy of YALFF applies this rule as follows: each k-mer of a read is searched in the dictionary and each mismatch makes the corresponding quality score untouchable, that is, it is sufficient to have one non concordant base in one of the k-mers to maintain the corresponding quality value unchanged. If all the k-mers are concordant with the reference for a particular base the associated quality is set to a default value (Fig. 1).

**Fig. 2:**
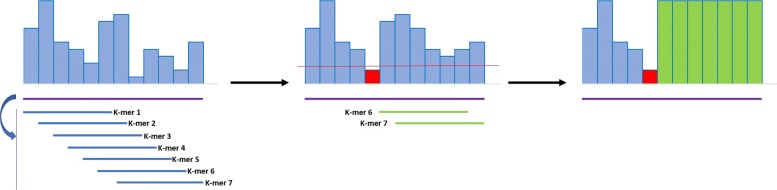
The threshold excludes k-mers 1 to 5 to be skipped by the algorithm

**Fig. 3:**
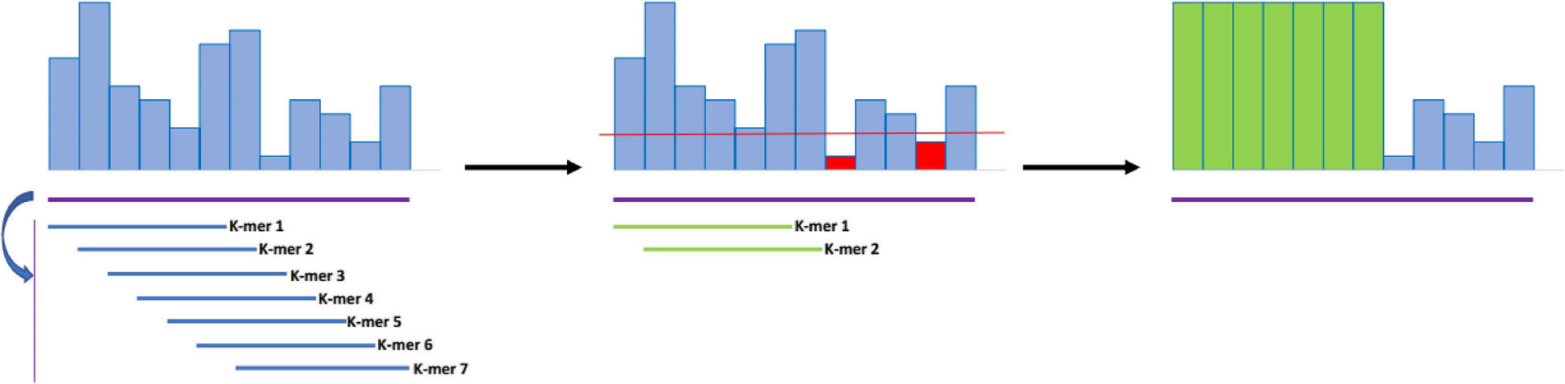
Example showing the threshold mechanism introduced to trim the low quality bases of a read. In this case only two k-mers are queried

This basic procedure has been modified to include a threshold for the quality scores (Fig. 2). All the scores below this threshold are maintained regardless of the outcome of the dictionary search. Such caveat is necessary to enforce YALFF to ignore very low quality k-mers. A very low quality base excludes all the k-mers covering that base as shown in Fig. 2 where the first five k-mers are skipped because of the single bad quality at position 5. The effect of having very low quality values will imply that all the k-mers containing one or more base with high probability of being incorrect are skipped. This also works as a trimming mechanism as shown in Fig. 3 where the tail of the read is left untouched.

**Fig. 4:**
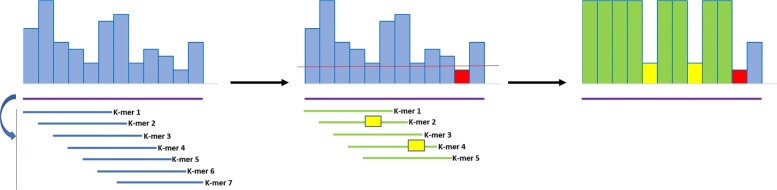
An example of quality smoothing by YALFF including both mismatches with the k-mers DB and low quality values

Figure 4 displays the whole mechanism including both mismatches with the k-mers DB and low quality values. The threshold should be chosen depending on if it is necessary to avoid as much distortion as possible or if compression is considered much more important. As a rule of thumb a higher threshold maintains more quality values unchanged but this leads to an increase in the entropy of the output file. It is advisable to plot an histogram of the distribution of quality values to choose the threshold accordingly. A good value found for this study was a quality value equal to the character ’ (apex) which corresponds to a probability of error of 0.25119.

According to other studies we selected the parameter *k*=32 [11, 12]. The k-mers should be long enough to ensure that the number of all possible k-mers is much larger than the number of unique k-mers in the genome, so as to ensure incidental collisions between unrelated k-mers are rare. Also, k-mer length should ideally be a multiple of four, since a 4bp length DNA sequence can be represented by a single byte. A 32-mer satisfies these constraints [11, 12]; it is represented by a single 64-bit integer, with a relatively low probability of containing more than one sequencing error with Illumina sequences, as well as resulting in few k-mer collisions.

### Implementation details

**Fig. 5:**
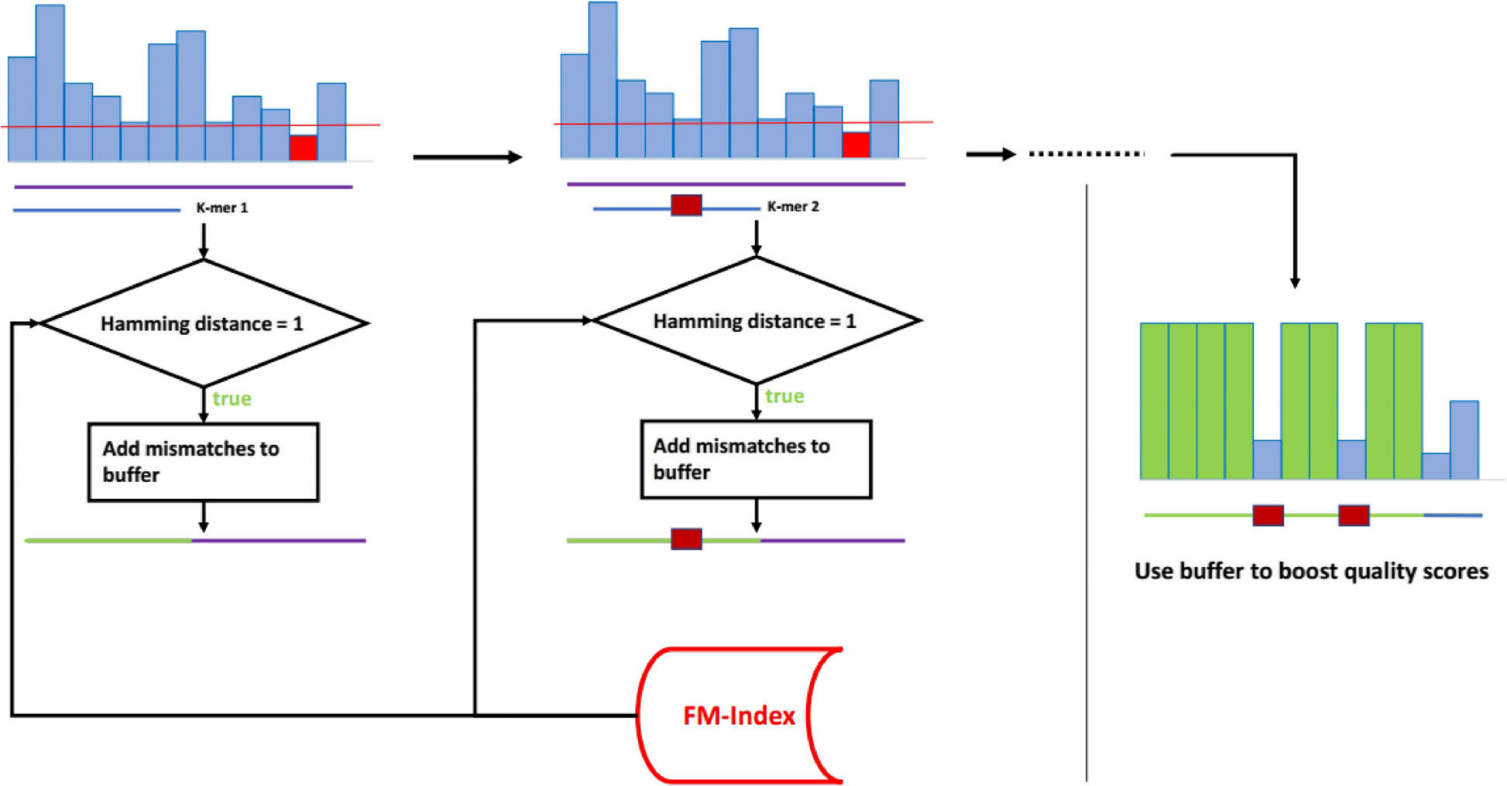
An overview of YALFF’s inner structure

YALFF is written in C/C++. The C parts are from BWA. In particular, the source code of the aln utility has been recycled to handle the query operations during smoothing. The FM-indexed version of a dictionary string is obtained through the index command of BWA. This opportunistic choice was made to ensure a widespread adoption of YALFF. Because BWA is the recommended aligner in most applications, it is extremely probable that a user who wants to compress some datasets will already have some sort of indexed reference genome which can be used as a dictionary. The indexing procedure and the data produced can be shared between our software and BWA leading to less time and storage required. In Fig. 5 is shown an overview of YALFF.

Because each read can be processed independently from the others, YALFF can be easily parallelized using more than one thread. The smoothed FASTQ files in output are guaranteed to maintain the order of the records compared to the input. This is particularly useful with paired end reads where the relative position of each read gives the association between them and thus has to be maintained.

In addition to the strictly necessary query parameters such as the k-mer length, the maximum number of mismatches allowed, the trimming threshold and the quality value used as replacement for the concordant bases, YALFF also supports other options. For example, as a speed up, it is possible to replace an entire block of quality values above a certain threshold with the smoothed value without searching the reference, or loading the reference in shared memory. Please refer to https://github.com/yhhshb/yalff for a complete description of the available options.

## Results

Since YALFF is a compressor where the reconstructed (i.e. decompressed) quality values can be different from the original ones, it is of uttermost importance to assess the effect that these changes in the quality values have on downstream applications. In the scope of this paper, in line with other studies, we choose variant calling as it is crucial for clinical decision making and thus widely used.

### Datasets, pipeline and parameters

The dataset used in this study is a set of real reads (NA12878) from the *1000 Genomes Project*http://www.internationalgenome.org/data-portal/sample/NA12878 . Only the two paired end archives were used (namely SRR622461_1.filt.fastq.gz and SRR622561_2.filt.fast.gz) for the evaluation, while the third containing unpaired reads were discarded. All tests have been done from scratch using the two paired end reads to evaluate the tools, without using previous results from other papers in order to make the comparison as clear as possible. This dataset has been widely used for benchmarking in other papers, because the list of known SNPs is available and it can retrieved from ftp://ussd-ftp.illumina.com/2017-1.0/hg38.

The reference genome used during alignment, and as a dictionary string for smoothing, is the human genome reference FASTA file hg38.fa downloaded from http://hgdownload.cse.ucsc.edu/goldenpath/hg38/bigZips/. The FM-index of the human genome is computed only once, in about 1h, and then it can be used for multiple runs of quality compression and reads alignment. The genotyping pipeline is implemented as a single bash script which uses bwa mem for alignments, bcftools for SNP calling and vcfeval for evaluation. All scripts can be found at https://github.com/yhhshb/yalff/tree/master/scripts.

Although YALFF can be run on a normal laptop, as opposed to Quartz, for all tools all tests were performed on a 14 lame blade cluster DELL PowerEdge M600 where each lame is equipped with two Intel Xeon E5450 at 3.00 GHz, 16GB RAM and two 72GB hard disk in RAID-1 (mirroring).

In this study we compared YALFF with other alignment-free methods, e.g. Quartz and Leon, as well as with other methods that are not based on the sequence like: Illumina 8bin, Pblock, Rblock and QVZ. As reported in [11, 12] reference-based methods are the most promising in terms of SNPs detection, in fact only these methods are able to improve the genotyping accuracy w.r.t. to the original reads. The choice to include Leon instead of GeneCodeq is because the latter does not provide an open source license but only non-optimized pre-compiled executables are available. Leon, on the other hand, is completely open-source and its binaries are optimized for most use cases. It also uses a probabilistic de-Brujin graph generated from the reads in input for smoothing instead of a predefined reference, thus widening the scope of the comparison. Leon does not produce a FASTQ file by default and uses its own compressed format instead. The exact commands for each program are reported in the Additional file 1.

The result section below shows time measurements for each tool defined as the time to obtain the smoothed FASTQ file from the original.

### Genotyping Accuracy

The performance evaluation of the algorithms compares the number of retrieved SNPs from a smoothed file to the ground truth associated with the original dataset. Each set of variants (stored in the output VCF file) is compared against the consensus set of variants. The benchmarking tools output the following values.

- True Positives (T.P.): All those variants that are both in the consensus set and in the set of called variants.
- False Positives (F.P.): All those variants that are in the called set of variants but not in the consensus set.
- False Negatives (F.N.): All those variants that are in the consensus set but not in the set of called variants.

These values are used to compute the following three metrics:

- Recall: This is the proportion of called variants that are included in the consensus set; that is, *R*=*T*.*P*./(*T*.*P*.+*F*.*N*.),
- Precision: This is the proportion of consensus variants that are called by the variant calling pipeline; that is, *P*=*T*.*P*./(*T*.*P*.+*F*.*P*.).
- F-Measure: The harmonic mean of precision and recall; that is, *F*−*Measure*=2∗(*P*∗*R*)/(*P*+*R*)

In the first experiments we run all tools and test how the modified quality values influence the detection of SNPs. We use the above metrics to assess the performance with respect to the original unsmoothed FASTQ file.

**Fig. 6:**
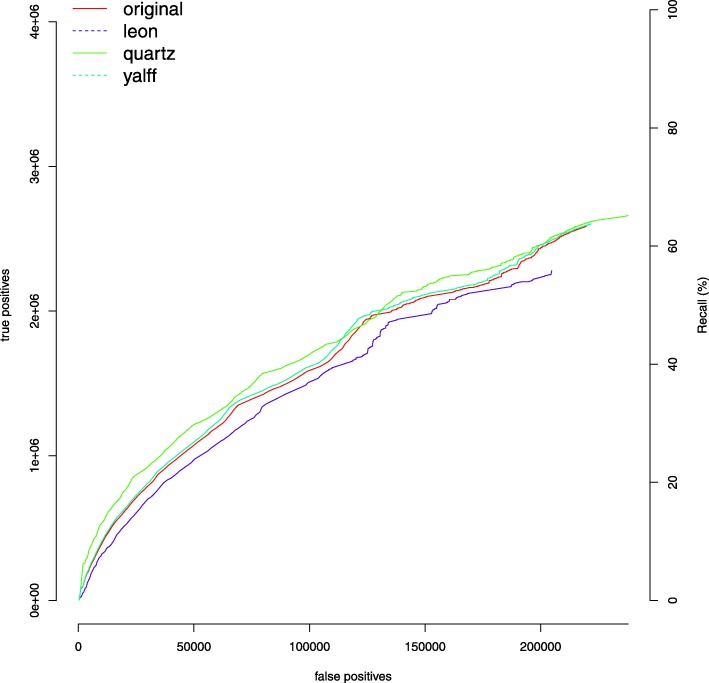
ROC curves of SNPs calling for various methods

Table 1 reports the results of these first experiments. The F-measure is a global indicator of the goodness of results, as it captures both precision and recall. If we compare the F-measures of all tools with respect to that of the original unsmoothed fastq, we can observe that the only methods that are able to improve this measure are Quartz and YALFF, whereas all other tools have lower F-measures. The F-measure improvement of Quartz is higher than YALFF and it is mainly due to the higher recall. A possible explanation is the fact that YALFF uses only k-mers from one reference genome, while the k-mers DB of Quartz is built from multiple genomes. Quartz shows the highest recall, that is, more SNPs are found, but at the expenses of the precision, in fact it exhibits the lowest precision and the highest number of false positives. If we consider the precision, the performance of Quartz degrades w.r.t. to the unsmoothed file, while YALFF is closer to it. High values of precision are reported also for Pblock, Rblock and Illumina 8bin, but in these cases the recall decreases. Overall, only Quartz and YALFF are to improve genotyping accuracy in terms of F-measure. However, YALFF produces very few false positive SNPs, as opposed to Quartz. This is a desirable characteristic especially in sensitive application such as health care or cancer analysis. Similar observations can be deduced from the ROC curves in Fig. 6. This Figure reports the number of true positives as a function of the false positives, and it includes for completeness the recall rate. More ROC curves can be found in the Additional file 1.

**Table 1:** Comparison of various metrics T.P., F.P., F.N., Precision, Recall and F-Measure in SNP calling between different tools

Smoothing algorithm	T.P.	F.P.	F.N.	Precision	Recall	F-Measure
None (original files)	2588159	219803	1493731	0.9217	0.6341	0.7513
YALFF	2603620	221368	1478264	0.9216	0.6378	0.7539
Quartz	2661218	237820	1420672	0.9180	0.6520	0.7624
Leon	2278517	204803	1803366	0.9175	0.5582	0.6941
Illumina 8bin	2546518	216128	1535370	0.9218	0.6239	0.7441
Pblock p =2	2574111	218405	1507773	0.9218	0.6306	0.7489
Pblock p =4	2558612	216995	1523273	0.9218	0.6268	0.7462
Rblock t =1.1	2550179	216738	1531706	0.9217	0.6248	0.7447
Rblock t =1.15	2526704	215721	1555181	0.9213	0.6190	0.7405
QVZ 0.6	2588704	225730	1493180	0.9198	0.6342	0.7507
QVZ 0.8	2588773	223210	1493112	0.9206	0.6342	0.7510

### Timing and RAM

We also compared the methods in terms of computing resources required for smoothing. The time measurements are the real wall clock times given by the time command on POSIX systems. Because all the tools where given one processor to perform their tasks the real wall clock time and the sum of user and sys times were comparable. All tools were tested on uncompressed FASTQ files because both Quartz and Leon don’t support compressed I/O. It must be noted that YALFF relies on the system pipe mechanism for read/write operations and can be used on compressed archives simply by command concatenation. The I/O operations and throughput handling are left to the pipe mechanism leading to a much more friendly experience.

**Fig. 7:**
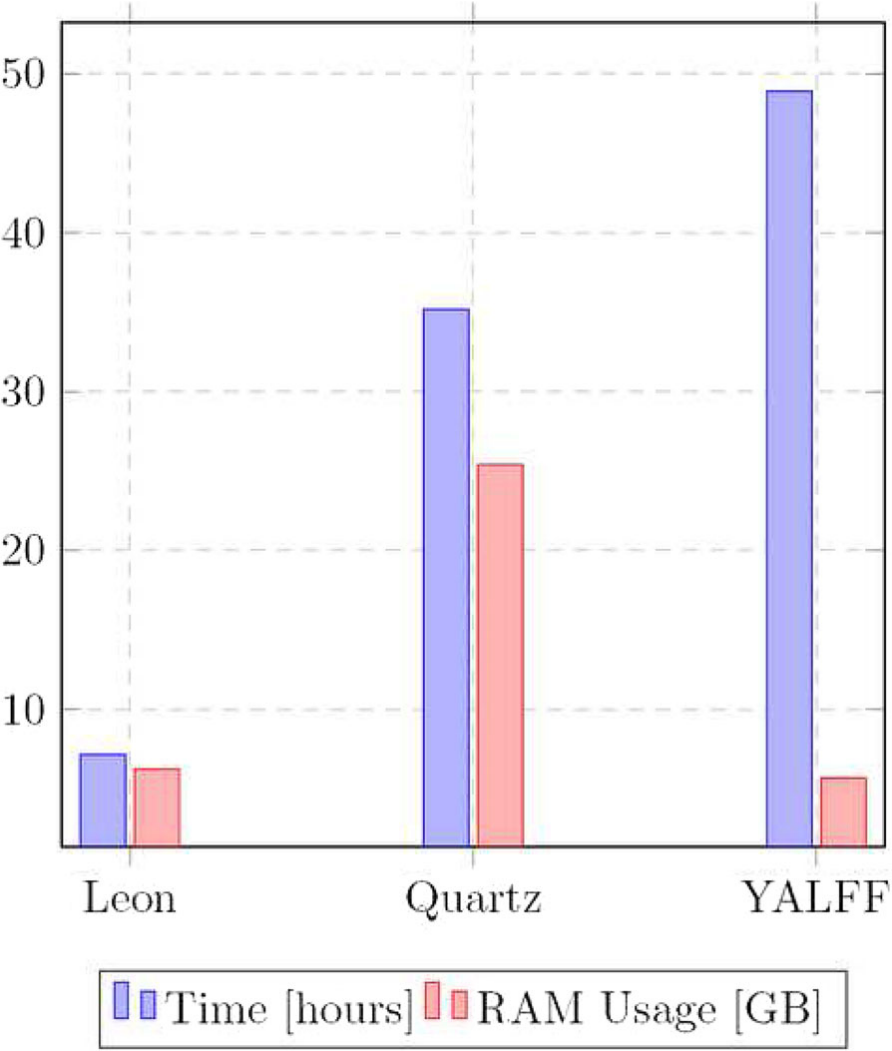
Histogram showing the total execution time in hours and peak RAM usage of the different programs

The fastest tools are those based only on the quality values, like Illumina 8bin, Pblock, Rblock and QVZ. They all require similar computing resources of about few GBs of RAM and 40 to 60 min for the execution. On the other hand, the methods that process also the sequence, like Quartz, YALFF and Leon, are more computationally demanding. Figure 7 shows a graphical representation of time and memory measures for Quartz, YALFF and Leon. In terms of execution times YALFF is the slowest of the three, with computing times comparable to Quartz, but not as fast as Leon. Leon on the other hand is the fastest, but it is also the least accurate tool with the worst precision and recall. YALFF despite being the slowest it requires less memory, only 5.7 GB of RAM, whereas Leon and Quartz need 6.3 GB and 25.4 GB respectively. Thus YALFF is the only one that can achieve good accuracy on SNPs calling and it can be used on a desktop computer, without relying on expensive hardware.

**Fig. 8:**
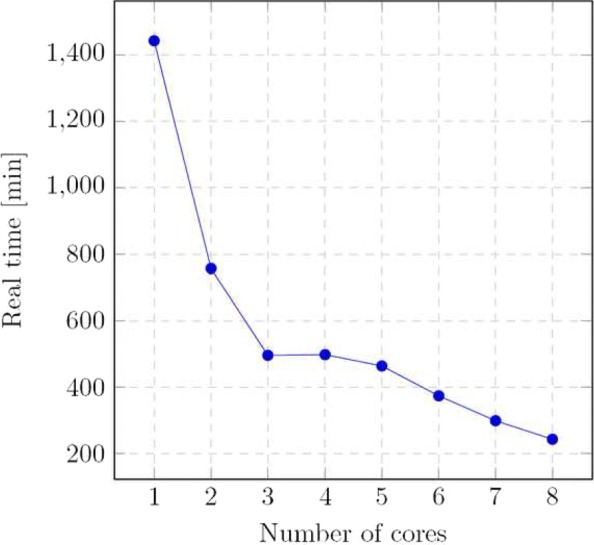
Time taken by YALFF to smooth a FASTQ file as a function of the number of cores

We tested also the scalability of YALFF. In Fig. 8 are reported the computing time of YALFF to smooth a single FASTQ file as a function of the number of cores used. To be able to make those scalability measures as reliable as possible each round has the number of cores allocated by a server supervisor so that no additional idle cores are present at each run. Both input and output streams use uncompressed files to make the plot comparable with the others. The optimal number of cores seems to be 3 or 4 but it strongly depends on the secondary storage device used and its characteristics. Using an SSD allows for better throughput and better core utilization. In summary YALFF can be easily parallelized to speed up smoothing.

### Compression

In this section we evaluate the compression ratios between the original uncompressed files and the compressed ones, while varying the smoothing method. To have a better overview of the compression ratios we used three lossless compressors, two widely used tool as gzip and bzips, and a more advanced one LZMA.

The results are shown in Table 2. As expected the smoothed files are more compressible that the original FASTQ. Also, LZMA achieves better compression ratios than gzip and bzip on all tests. In terms of compression ratio, the methods based on the sequence are able to achieve better compression. Among the methods based only on the quality values, Rblock appears to be the best one. If we consider all smoothing methods we can observe that YALFF has the best compression ratios, outperforming all other tools, irrespective of the compressor used.

**Table 2:** Compression ratio for the different smoothing tools and compressors. The ratio is defined as $\frac {\text {uncompressed size}}{\text {compressed size}}$ where the uncompressed size is 42GB

Smoothing algorithm	gzip	bzip2	xz (LZMA)
None (original files)	4.617	5.152	5.918
YALFF	7.147	7.633	9.186
Quartz	6.925	7.349	8.827
Leon	7.098	7.551	8.988
Illumina 8bin	6.054	6.742	7.819
Pblock p =2	5.373	5.966	7.011
Pblock p =4	6.052	6.647	7.671
Rblock t =1.1	6.285	6.859	7.941
Rblock t =1.15	6.675	7.250	8.443
QVZ 0.6	4.776	5.533	6.395
QVZ 0.8	4.778	5.510	6.366

### YALFF parameters

In this section we evaluate the impact of the parameters of YALFF. We recall that YALFF has three parameters: k for the length of k-mers, the lower quality threshold (L.T.) for trimming and the higher quality threshold (H.T.) to speed-up the smoothing. The thresholds L.T. and H.T. are expressed using the Phred quality representation, that for Illumina spans between 0 (poor quality) to 41 (high quality). In the previous experiments we used as default values k =32, L.T. =6 and H.T. =40. In Table 3 we report the performance of YALFF for various parameters.

**Table 3:** The impact of the parameters of YALFF for various metrics T.P., F.P., F.N., Precision, Recall, F-Measure, Compression (LZMA) and Time (min.)

Parameters										
k	L.T.	H.T.	T.P.	F.P.	F.N.	Prec.	Recall	F-M.	Compr.	Time
16	6	40	2659170	276596	1422714	0.9058	0.6515	0.7579	10.107	11850
32	6	40	2603620	221368	1478264	0.9216	0.6378	0.7539	9.186	2934
48	6	40	2588254	220176	1493631	0.9216	0.6341	0.7513	5.936	509
32	0	40	2626957	253747	1454928	0.9119	0.6436	0.7546	9.181	2657
32	3	40	2603891	221696	1477993	0.9215	0.6379	0.7539	9.113	2315
32	12	40	2601463	219417	1480421	0.9222	0.6373	0.7537	8.813	2239
32	3	30	2616530	225716	1465356	0.9206	0.6410	0.7558	9.597	565
32	3	35	2612145	223372	1469743	0.9212	0.6399	0.7552	9.429	848
32	3	37	2609021	222494	1472866	0.9214	0.6392	0.7548	9.115	1279

The most important parameter is the length of the k-mers. If k is small, e.g. k =16, there is a small improvement in terms of F-measure and compression, however this comes at the expenses of the computing time that increases substantially. On the other hand, if k =48, the running time decreases, but also the compression decreases. We choose k =32 as the best compromise between compression and computing time. The lower threshold (L.T.) is used for trimming low quality values, that are not boosted by YALFF. If this threshold is not applied (L.T. =0) the precision decreases. If higher values are used (L.T. =12) the precision increases, but the compression decreases. We choose L.T. =6 as a trade off between trimming and compression. The higher quality threshold (H.T.) can be used to speed-up the computation by boosting all quality values above H.T. If we use H.T. =30 the computation time decreases considerably, with a small reduction of precision. However, if time is not a constraint and precision is most important, we suggest to use high values of H.T.

## Discussion

The low compressibility of quality values is one of the main problem of sequencing reads compression. Several lossy smoothing strategies have been proposed, all with the intent to improve compressibility without altering the information carried by quality value for downstream analysis. Here, we propose YALFF, a tool that smooths quality scores based on a dictionary of k-mers from a reference genome. The YALFF smoothing algorithm can achieve low distortion of the processed datasets with a small degradation of precision during SNP calling, but with an overall improvement of F-measure. We developed this program with consumer application of genome sequencing in mind. For example, one of the current hot topic is personalized medicine, which requires huge databases to store as many genomic information as possible and new methods to allow common users to share their genetic code. New compression programs needs to be developed to tackle these problems. Tools with reduced memory consumption, like YALFF, to be executed on commodity computers, will enhance the sharing of sequencing data.

Unfortunately, YALFF is not perfect and it can be further improved. Its main flaw is the time inefficiency compared to e.g. Quartz or Leon. Using a compressed data structure as a dictionary can compromise cache efficiency. The main question which needs to be investigated further is if it is possible to develop a compressed dictionary with good locality properties.

## Conclusions

In this work, we have presented YALFF, a lossy FASTQ smoother which uses a dictionary of k-mers that are compressed with a BWT. YALFF is able to reduce the entropy of quality scores by smoothing leading to improved compressibility of FASTQ files w.r.t. to other popular tools. The succinct dictionary allows YALFF to run on consumer computers with only 5.7 GB of RAM, as opposed to Quartz that requires large amount of memory. The smoothing algorithm of YALFF can improve the genotyping accuracy, in terms of F-measure, when compared with the unsmoothed FASTQ, and it can also reduce the number of false positive, w.r.t. Quartz. In summary YALFF produces smoothed FASTQ that are highly compressible, while maintaining high accuracy on genotyping and using less resources.

## Additional file

Additional file 1 Supplementary Material. (PDF 303 kb)

## Abbreviations

BWT: Burrows-wheeler transform
NGS: Next-gen sequencing
SNP: Single nucleotide polymorphisms
YALFF: Yet another lossy fastq filter
