# Supplementary material for: Better quality score compression through sequence-based quality smoothing
Source: BMC Bioinformatics. 2019 Nov 22;20(Suppl 9):302. doi: 10.1186/s12859-019-2883-5 (PMC6873394; doi:10.1186/s12859-019-2883-5)
Supplement: Supplementary file 1 — Supplementary Material. (PDF 303 kb) [file 12859_2019_2883_MOESM1_ESM.pdf]

Supplementary Materials

How to run quality compression tools

In the following we report the exact commands for each compression program used in paper:

Quartz:

```
./quartz dec200.bin.sorted 'S' 1 0 <Input file 1> <Input file 2>
```

Leon:

```
./leon -file <Input file> -c -nb-cores 1
```

Illumina:

```
./il8b convert <Input file> -o <Output file>
```

Pblock:

```
./pblock <Input file> <p>
```

Rblock:

```
./rblock <Input file> <r>
```

QVZ:

```
awk ’!(NR%4)’ <Input fastq file> > <quality file>
./qvz -c 3 -r <r> -u <smoothed uncompressed qualities> <quality file> <smoothed compressed qualities>
```

YALFF:

YALFF Parameter k (16, 32, 48):

```
cat <Input> | ./yalff -d hg38.fa -k 16 -b \' -t 1 > output.fastq
cat <Input> | ./yalff -d hg38.fa -k 32 -b \' -t 1 > output.fastq
cat <Input> | ./yalff -d hg38.fa -k 48 -b \' -t 1 > output.fastq
```

YALFF Lower Threshold (Phred values: 0 = '!', 3 = '$', 12 = '-'):

```
cat <Input file> | ./yalff -d hg38.fa -b \! -t 1 > output.fastq
cat <Input file> | ./yalff -d hg38.fa -b \$ -t 1 > output.fastq
cat <Input file> | ./yalff -d hg38.fa -b \- -t 1 > output.fastq
```

YALFF Higher Threshold (Phred values: 30 = '?', 35 = 'D', 37 = 'F'):

```
cat <Input file> | ./yalff -d hg38.fa -g \? -t 1 > output.fastq
cat <Input file> | ./yalff -d hg38.fa -g D -t 1 > output.fastq
cat <Input file> | ./yalff -d hg38.fa -g F -t 1 > output.fastq
```

More ROC curves

Here, we report other ROC curves. In Figure 1 we compare quality compression methods that are not based on the sequence, and in Figure 2 we evaluate YALFF for various parameters.
ROC curves are usually represented with true positive rate vs false positive rate. However, if different VCF files contain a different number of calls, the direct comparison of these rates, and of AUCs, is not possible. There are a several difficulties involved with computing these rates for different VCF files and different variant calling datasets [1], and it has been shown that the AUC is not the best indicator [2]. Thus, in line with [3], in the following figures we report the absolute count of true positive and false positive, as well as the recall rate.

Figure 1

ROC curves of SNPs calling for various quality compression tools not based on the sequence

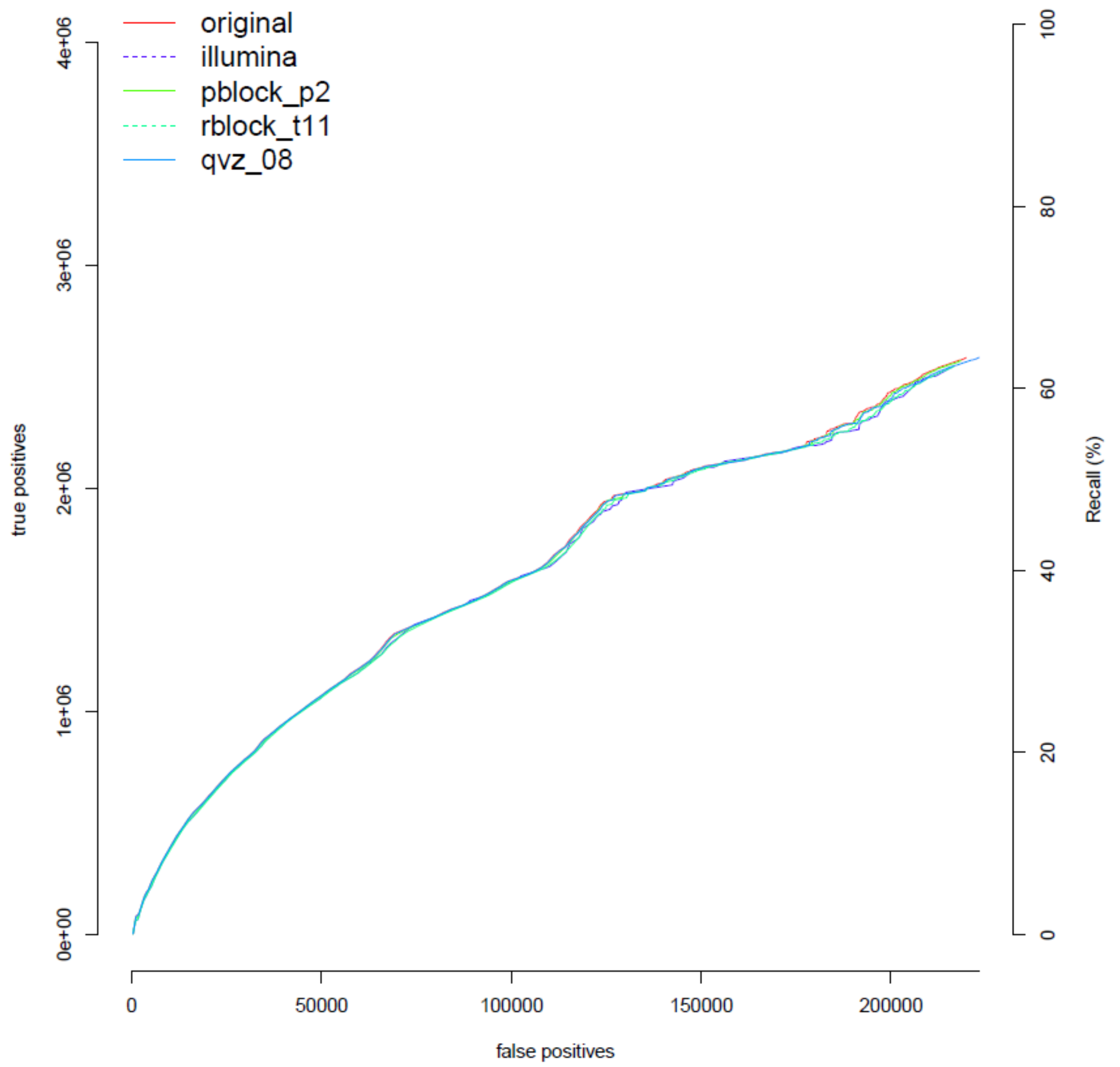

Figure 2

ROC curves of SNPs calling for YALFF with various parameters

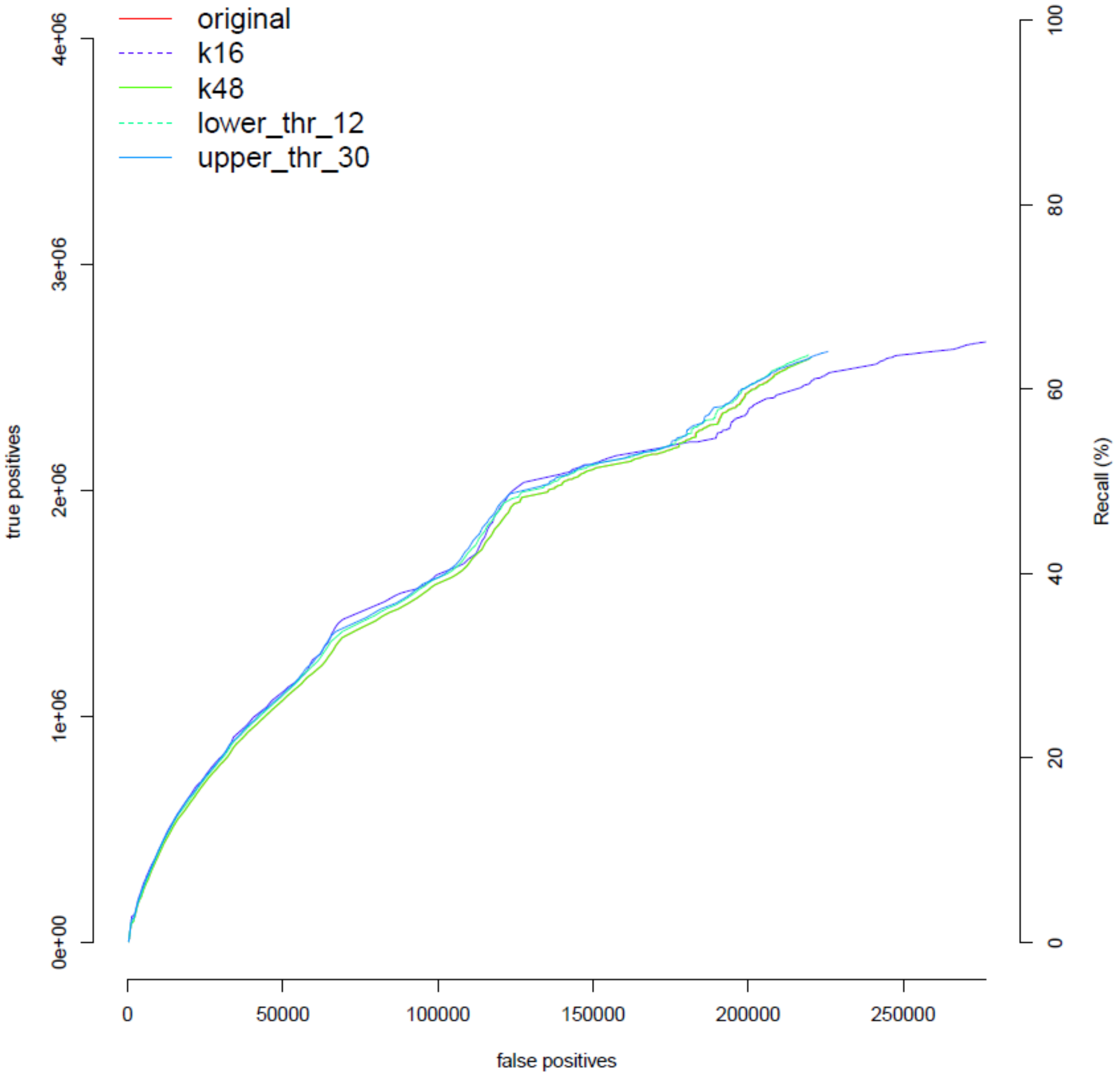

References

[1] Peter Krusche, Len Trigg, Paul C. Boutros, Christopher E. Mason, Francisco M. De La Vega, Benjamin L. Moore, Mar Gonzalez-Porta, Michael A. Eberle, Zivana Tezak, Samir Lababidi, Rebecca Truty, George Asimenos, Birgit Funke, Mark Fleharty, Brad A. Chapman, Marc Salit, Justin M Zook, and the Global Alliance for Genomics and Health Benchmarking Team. Best Practices for Benchmarking Germline Small Variant Calls in Human Genomes.
bioRxiv pre-print: https://www.biorxiv.org/content/biorxiv/early/2018/05/24/270157.full.pdf

[2] Lobo, J. M., Jiménez-Valverde, A. and Real, R. (2008), AUC: a misleading measure of the performance of predictive distribution models. Global Ecology and Biogeography, 17: 145-151.
doi:10.1111/j.1466-8238.2007.00358.x

[3] Malysa, G., Hernaez, M., Ochoa, I., Rao, M., Ganesan, K., Weissman, T.: QVZ: lossy compression of quality values. Bioinformatics (Oxford, England) 31(19), 3122-3129 (2015).
doi:10.1093/bioinformatics/btv330
